# Prevalence of feline upper respiratory tract pathogens and risk factors for clinical disease and final outcomes in an RSPCA shelter in Queensland, Australia

**DOI:** 10.1002/vro2.70001

**Published:** 2024-12-06

**Authors:** Uttara Kennedy, Mark Stevenson, Mandy Paterson, Susan Jaensch, Doug Hayward, Nicholas Clark

**Affiliations:** ^1^ School of Veterinary Science University of Queensland Gatton Queensland Australia; ^2^ Royal Society for the Prevention of Cruelty to Animals Queensland Wacol Queensland Australia; ^3^ Melbourne Veterinary School University of Melbourne Melbourne Victoria Australia; ^4^ Vetnostics, Macquarie Park New South Wales Australia

## Abstract

**Introduction:**

Feline respiratory tract infection poses a serious challenge in animal shelters. Potential risk factors include pathogens introduced through animals entering the shelter. We aimed to determine the proportion of animals shedding feline upper respiratory tract (URT) pathogens at the time of entry and to assess how this contributed to the burden of clinical disease and final outcomes.

**Methods:**

Oropharyngeal and conjunctival swabs were collected from incoming cats over 11 months and tested using real‐time PCR. The prevalence and distribution of pathogens were reported; causal associations with clinical disease and shelter outcomes were assessed using Bayesian generalised regression models.

**Results:**

On admission, 43% (*n* = 86) cats were shedding one or more pathogens (feline herpes virus, feline calici virus, *Mycoplasma felis*, *Chlamydia felis* and *Bordetella bronchiseptica*). Shedding was somewhat associated with subsequent clinical disease but not with risk of euthanasia. Animals placed into foster care were less likely (odds ratio [OR] 0.27, Bayesian credible interval [CI] 0.09‒0.78) and those enrolled into behavioural rehabilitation programmes were more likely to develop disease (OR 5, CI 2.4‒11). Kittens had a delayed time to onset of disease (daily hazard 0.39, CI 0.13‒1.2). Geriatric animals (OR 4.1, CI 1.8‒10) and those with comorbidities (OR 8.8, CI 3.5‒25) were most likely to be euthanased.

**Conclusions:**

While a substantial proportion of animals were shedding pathogens on entry, animal characteristics (age and behaviour) and shelter operations (foster care) were more important in impacting the shelter's burden of clinical feline URT disease.

## INTRODUCTION

Animal shelters are frequently burdened with controlling infectious diseases. Factors such as high population densities, poor air circulation and occasional comorbidities (such as malnourishment) at time of entry^1^, ^2^, ^3^, ^4^, ^5^ directly contribute to higher stress levels, lower immunity and increased risk of disease spread.^6^, ^7^ Feline upper respiratory tract (URT) disease is a particularly challenging disease worldwide.^5^, ^8^ Infection leads to a protracted length of stay (LOS) in the shelter, impeding optimal use of the shelter's resources and space,^2^, ^3^, ^5^, ^9^, ^10^ and an increased rate of euthanasia of seriously affected cats.^11^ The pathogens implicated in the disease include feline herpes virus (FHV‐1), feline calici virus (FCV), *Mycoplasma felis*, *Chlamydia felis* and *Bordetella bronchiseptica*. Clinical signs include nasal discharge, sneezing, dyspnoea, conjunctivitis, ocular discharge and ulcerations of the cornea, tongue, gums or nasal planum.^9^

While spread can be mitigated through modifiable husbandry and management strategies, such as enhanced enrichment and size of enclosures or positive handling,^5^, ^12^, ^13^ it is challenging to quantify the effectiveness of such systems. An assessment of whether the shelter's burden of disease is due to incoming animals bringing pathogens into the shelter, or through propagation within the shelter, can inform such shelter operations.

Previous prevalence studies have reported diverse rates of infection. One study found that 29% of cats were shedding at least one of the five pathogens on entry into a shelter in Canada,^1^ and another study of five shelters in the USA reported a maximum of 38% of shedding cats.^5^ The incoming prevalence of viral mono‐infection in a Belgian shelter was 20% (95% credible interval [CI]: 15.7%–25.1%) (FHV) and 33% (95% CI: 27.8%–38.8%) (FCV), while co‐infection was 10% (95% CI: 6.8%–14%).^14^

Our study aimed to quantify feline URT pathogen introduction into an Australian shelter. Our objectives were to (1) report on the proportion of incoming animals shedding URT pathogens at time of entry into the shelter, (2) determine if shedding on entry was associated with disease incidence and (3) identify modifiable environmental and population health factors associated with important shelter outcomes (time to onset of disease, disease severity and endpoints such as adoption and euthanasia).

## METHODS

### Study setting and subjects

The Royal Society for the Prevention of Cruelty to Animals, Queensland (RSPCA Qld) is Queensland's leading animal welfare charity. The charity received 9633 cats statewide in the 2021‒2022 financial year, of which over 2800 were received by the charity's largest urban shelter (Wacol, Qld).^15^ A convenience sample of oropharyngeal and conjunctival samples was collected from selected cats entering the Wacol shelter between 2 June 2022 and 19 April 2023 (inclusive).

Animals entered the shelter via the shelter's ambulances, owners surrendering their pets or cats seized by the shelter's inspectorate as a result of cruelty investigations. All incoming cats underwent general physical examination within 24 h of entry. Cats needing veterinary treatment were housed in medical wards (single story metal cages, area 6.2 ft^2^), while animals deemed suitable for adoption were housed in single or double‐story individual cages (6.2–12 ft^2^) or large rooms for group housing. Food and water were placed at the furthest corner from litter areas; appropriate enrichment was placed in cages as per individual needs (overhanging towels, scratching posts and toys). A typical enclosure and details of the cleaning protocols are described in Supporting Information S1.1. Animals exhibiting high levels of stress, anxiety or withdrawal were sent to a rehabilitation ward under direct care of dedicated rehabilitation staff. The cats were visually checked multiple times a day and those exhibiting signs of disease, pain or discomfort were flagged with veterinary staff. If clinical signs of URT infection were observed, they were transferred to ‘cat‐flu’ isolation wards. Veterinary observations were entered in each animal's electronic veterinary record. Select animals were sent to foster depending on availability of carers and the animals’ medical and behavioural needs.

### Sample collection

On entry, pre‐determined criteria were used to select animals that were newly incoming (that is not transferred from another RSPCA shelter) and over the age of 6 weeks (to ensure appropriate size for swab insertion, Supporting Information S1.2). Sample size was determined by power analysis, using prior knowledge of expected disease prevalence and risk factor effect sizes. Cats were gently restrained and swabbed within 24 h of entry, as per an approved standard operating protocol (Supporting Information S2.1). Each cat had one dry cotton swab gently rolled in the caudal oropharynx, avoiding contact with the tongue and a second dry cotton swab rolled in the ventral fornix of one or both eyes.^16^ Immediately after collection, a live attenuated vaccination against FHV‐1, FCV and feline panleukopenia virus (Companion F3, MSD Animal Health) was administered. Swabs were initially stored at ‒20°C on site, transferred to ‒80°C storage within 10 days of collection‐ and then transported on dry ice (via 24‐h courier) to a commercial laboratory for real‐time PCR assays (Supporting Information S2.2).

The following data were retrieved for each animal—unique animal identifier assigned at the time of entry, date of sample collection, age (as per registered microchip if present or estimated by shelter staff from general appearance and dentition), sex, source and neuter status. Age was further converted into a factored variable—animals aged less than 4 months (kitten), animals aged between 4 months and 8 years (adult) and animals greater than 8 years (geriatric). These categories were chosen based on automated classification by the shelter's software, which is used to inform shelter procedures and protocols. Once the cat had permanently left the shelter, data were collected on its final outcome, whether fostering or behavioural intervention had occurred, date of exit, number of housing locations and LOS. The presence of comorbidities and time to onset of disease were manually annotated from veterinary records using a previously defined set of criteria.^17^ The exposure and outcome variables are described in Supporting Information S3. A graded score (1‒4) was used to assign a disease severity index to each positive case based on its treatment protocol (Supporting Information S4).

### Statistical analyses

The prevalence and distribution of pathogens on entry were reported using descriptive analysis.

### Causal frameworks

**FIGURE 1:**
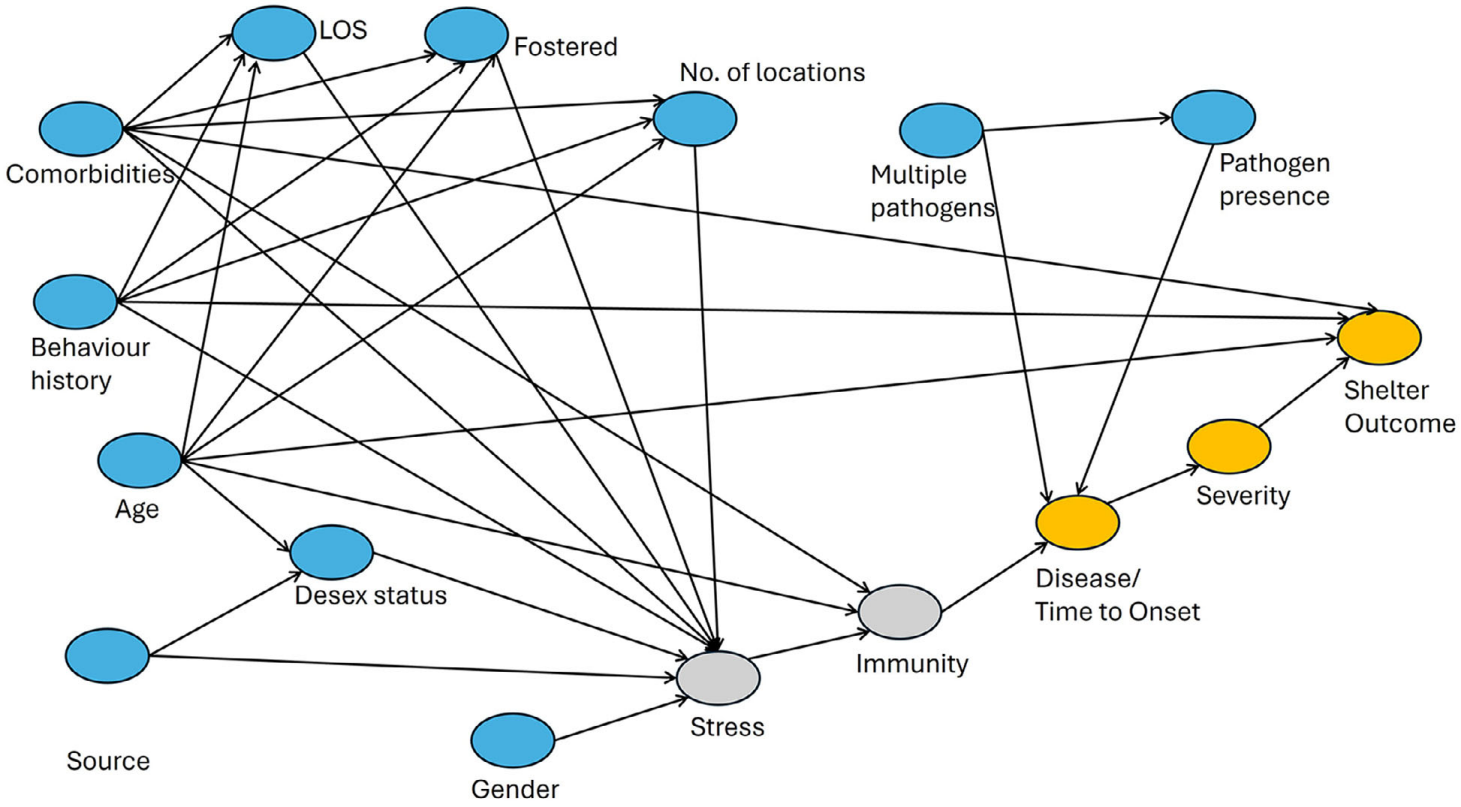
Direct acyclic graph informing regression models. Postulated causal relationships between exposure variables and final outcomes (upper respiratory tract disease/time to onset, severity and shelter outcome). Blue: exposure variables, grey: unmeasured variables, orange: outcome variables. LOS, Length of stay.

A directed acyclic graph (DAG) was developed, showing factors thought to be causally associated with disease presence, time to onset, severity and final outcome (Figure 1). The DAG was informed from published studies and the opinions of shelter clinicians. For each exposure‒outcome association, explanatory variables (‘sufficient adjustment sets’) that should be adjusted for, were determined using the do‐calculus method^18^, ^19^ implemented in DAGitty.^20^ Details of hypothesised explanatory variables are provided in Table 1.

**TABLE 1:** Exposure and outcome variables for cats.

Variable	Values	Type	
**Age**	Categorical	Exposure	
Kitten			15.6%
Adult			63.3%
Geriatric			21.1%
**Gender**	Binary	Exposure	
Male			58.5%
Female			41.5%
**Animal source**	Categorical	Exposure	
Stray			14.1%
Surrender			22.6%
Ambulance			50.7%
Other			12.6%
**Neuter status on entry**	Binary	Exposure	
Yes			34.2%
No			65.8%
**Pathogen presence**	Binary	Exposure	
Yes			43.2%
No			56.8%
**Coinfection**	Binary	Exposure	
Yes			14.1%
No			85.9%
**Foster status**	Binary	Exposure	
Yes			22.6%
No			77.4%
**Length of stay**	Numeric (days)	Exposure	Minimum 0, maximum 185 Median 16 IQR (3, 48)
**Comorbidities**	Binary	Exposure	
Yes			67.8%
No			32.2%
**Behavioural status**	Binary	Exposure	
Yes			18.6%
No			81.4%
**Number of housing locations**	Numeric	Exposure	Minimum 1, maximum 16 Median 4 IQR (2, 5)
**Final outcome**	Categorical	Outcome	
Adopted			55.8%
Euthanased			32.7%
Other			11.5%
**Feline upper respiratory tract clinical disease**	Binary	Outcome	
Yes			20.1%
No			79.9%
**Severity score**	Ordered factor	Outcome	
Grade 1			17.5%
Grade 2			32.5%
Grade 3			42.5%
Grade 4			7.5%

Abbreviation: IQR, interquartile range.

### Risk of developing clinical URT signs over time

Time‐to‐event analyses were carried out using the Kaplan‒Meier method.^21^ The outcome of interest was calculated as the time from entry to the date of the first observed clinical sign. Animals that died or left the shelter before developing clinical signs were treated as censored observations.

Cox proportional hazards regression models were developed based on the sufficient adjustment sets identified from our DAG. To verify the proportional hazards assumption, we plotted the scaled Schoenfeld residuals from each model as a function of follow‐up time. The proportional assumption was considered violated if a line of gradient zero could not be drawn between the 95% confidence intervals of the loess‐smoothed line of best fit.^22^ Survival analyses were carried out using the contributed survival package implemented in the R statistical software package version 4.3.1.^23^, ^24^ To demonstrate the difference in hazard of disease between covariate groups, population attributable fractions (PAFs) were calculated using the AF package.^25^

### Risk factors associated with the development and severity of clinical URT signs and with euthanasia

We fitted multiple Bayesian generalised (non‐)linear multivariable multilevel models with logit link (analogous to a logistic regression), using the brms interface in Stan for performing full Bayesian inference.^26^, ^27^ The first set of models used logistic regression to assess the association of various exposure variables on the presence of disease, the next set used ordinal regression.^28^ for effect on the severity of disease (an ordinal variable graded into four severity categories) and the final set assessed relationships with final outcome (adopted or euthanased).

The encoded priors, which were based on a previous study,^29^ were weakly informative and consistent with domain expertise, encouraging regularised posterior estimates (Supporting Information S5 and Figure S1). Posteriors were approximated by drawing samples using Markov chain Monte Carlo sampling through Hamiltonian Monte Carlo using Stan.^27^ The sampler used 1000 iterations as burn‐in, followed by 2000 iterations with four chains. Diagnostic trace plots were visually checked for stability of simulation results and model convergence. Summaries of posterior draws, including group‐level effects and population‐level effects, were obtained. The motivation for using Bayesian analysis was that it formally incorporates prior assumptions about expected variation and magnitude of potential effects; furthermore, it provides a more robust analysis in low‐power situations.^30^

Parameter estimation in a Bayesian framework does not return point estimates, but rather a posterior distribution of possible values for a parameter,^31^ with a ‘probability’ or ‘credible’ interval. In contrast to a frequentist (confidence) interval, which is interpreted in the context of repeated similar practice, the Bayesian interval is directly interpreted as having a high chance of containing the true value.^32^ Reported point estimates in the form of odds ratios (ORs) or hazard ratios were interpreted within the context of their CIs, which indicated where the majority of the posterior mass resided. When the posterior mass is mostly away from 1, there is a strong possibility of an association, even if there is uncertainty in the magnitude of this effect. The contributed marginal effects package in R was used to compute and plot predictions and marginal effects from each model.^33^

## RESULTS

**FIGURE 2:**
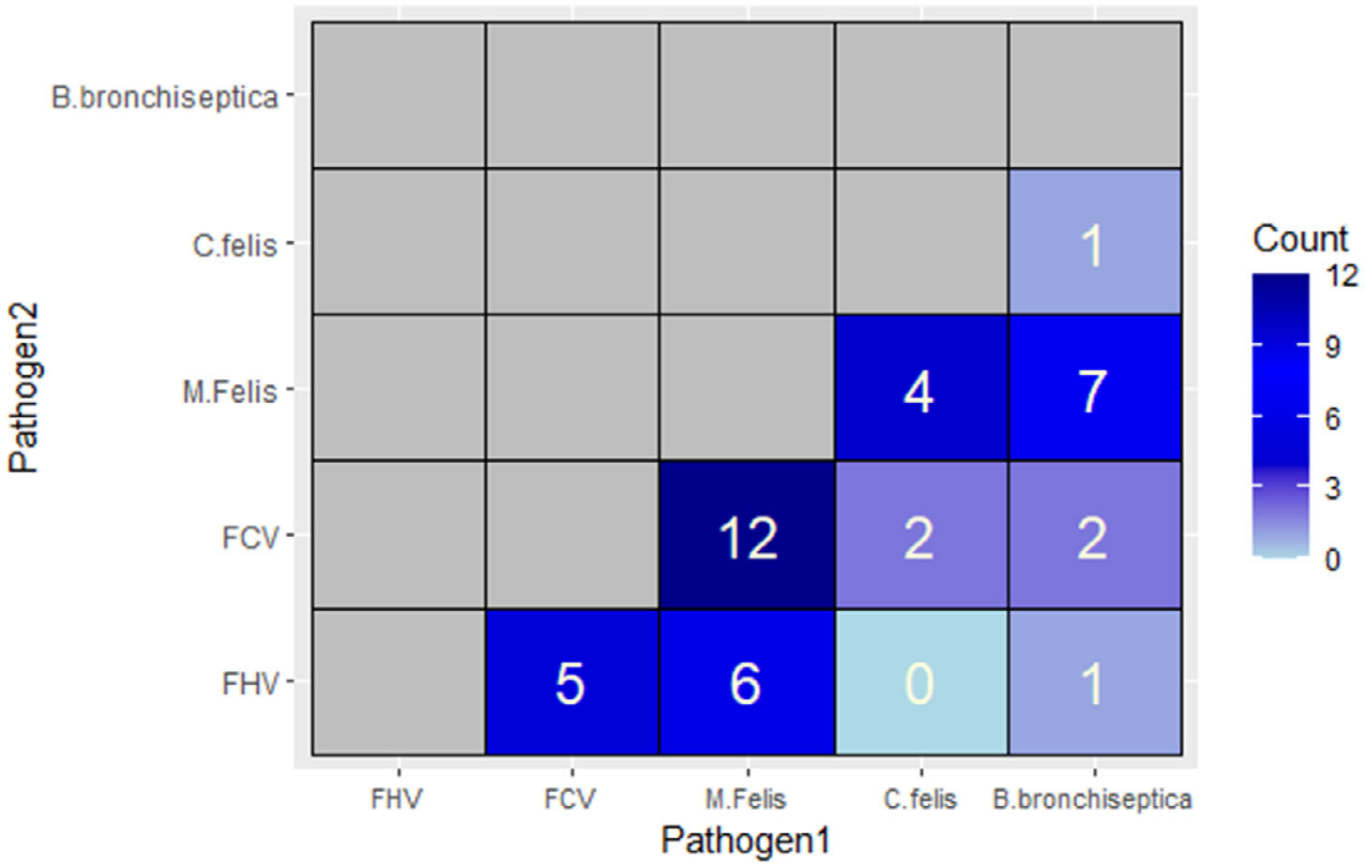
Co‐occurrence matrix for pathogens recovered by real‐time PCR testing. Note that some animals were simultaneously infected by more than one pathogen.

A total of 199 animals were included in the study. All animals showed sufficient sample adequacy on PCR testing. Forty‐three percent (*n* = 86) were positive for at least one pathogen on entry. Of these, 33% (*n* = 28) were co‐infected with more than one pathogen (Figure 2). None of the animals were positive for all five pathogens. Of the positive samples, 62% (*n* = 53), 36% (*n* = 31), 19% (*n* = 16), 15% (*n* = 13) and 8% (*n* = 7) were positive for *M. felis*, FCV, FHV, *B. bronchiseptica* and *C. felis*, respectively.

Just over half the animals (*n* = 101) were brought in via the internal ambulance service, while the remaining cats were surrendered to the shelter (*n* = 55), strays (*n* = 28), seized due to cruelty complaints (*n* = 11) or needed emergency boarding (*n* = 4). Overall, 57% were male (*n* = 114) while 41% were female (n = 81); four animals did not have their sex recorded. We found that 30% of the males and 41% of the females were neutered prior to entry. The recorded age range was between 1.2 months and 21 years, with a median age of 2 (Q1, 0.8; Q3, 5) years.

Of the 23% (*n* = 45) of the cats placed in foster, only three developed clinical URT disease after being placed in foster. Nineteen percent (*n* = 37) of the animals needed behavioural interventions due to anxiety, fear and stress. The median LOS was 16 (Q1, 3; Q3, 48) days; however, 12 animals stayed for more than 100 days, with one animal staying 185 days. The maximum number of locations was 16, with the median being 4 (Q1, 2; Q3, 5).

Positive outcomes such as being adopted (111 of 199), reclaimed (*n* = 15) or returned after emergency boarding (*n* = 4) were seen in 65% of cases. The remainder were euthanased (*n* = 65) or died during their stay in the shelter (*n* = 4).

### Risk of developing clinical URT signs over time

**FIGURE 3:**
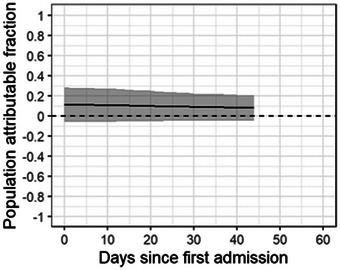
Population attributable fraction for animals needing behavioural rehabilitation. The solid line indicates the increase in clinical upper respiratory tract disease seen in the first 50 days post‐admission if the entire cohort of animals need behavioural rehabilitation.

Animals needing behavioural rehabilitation had 1.74 (95% Bayesian CI 0.85‒3.43) times the daily hazard of developing disease compared with cats that did not. The adjusted PAF for behavioural rehabilitation (proportion of cases that were attributable to need for behavioural rehabilitation) was 0.11 (95% CI ‒0.06 to 0.27) (Figure 3).

The daily hazard for kittens was 0.39 (95% CI 0.13‒1.2) times that of older animals. The remaining explanatory variables showed wide hazard estimates either side of 1, resulting in uncertainty around the magnitude and direction of their effects (Table 2). The median time to onset was 33 days; the longest time being 44 days.

**TABLE 2:** Direct acyclic graph‐guided Cox proportional hazards regression model outputs for covariates associated with time to onset of clinical upper respiratory tract disease.

Covariate (*n*)	Hazard ratio (adjusted)	Hazard ratio (unadjusted)
**Gender**		
Male (*n* = 114) Female (*n* = 81)	Ref 0.62 (CI 0.32, 1.22)	Ref 0.66 (CI 0.34, 1.29)
**Comorbidities**		
N Y	Ref 1.23 (CI 0.60, 2.52)	Ref 1.22 (CI 0.62, 2.40)
**Pathogen presence**		
N Y	Ref 1.27 (CI 0.61, 2.65)	Ref 1.65 (CI 0.89, 3.08)
**Behaviour history**		
N Y	Ref 1.74 (CI 0.88, 3.43)	Ref 2.12 (CI 1.13, 3.98)
**Multiple infections**		
N Y	Ref 1.42 (CI 0.55, 3.62)	Ref 1.75 (CI 0.81, 3.81)
**Age**		
Kitten Adult Geriatric	0.39 (CI 0.13, 1.16) Ref 0.81 (CI 0.31, 2.16)	0.34 (CI 0.12, 0.96) Ref 0.98 (CI 0.41, 2.37)

Abbreviation: CI, confidence interval.

### Risk factors associated with presence and severity of clinical disease

**FIGURE 4:**
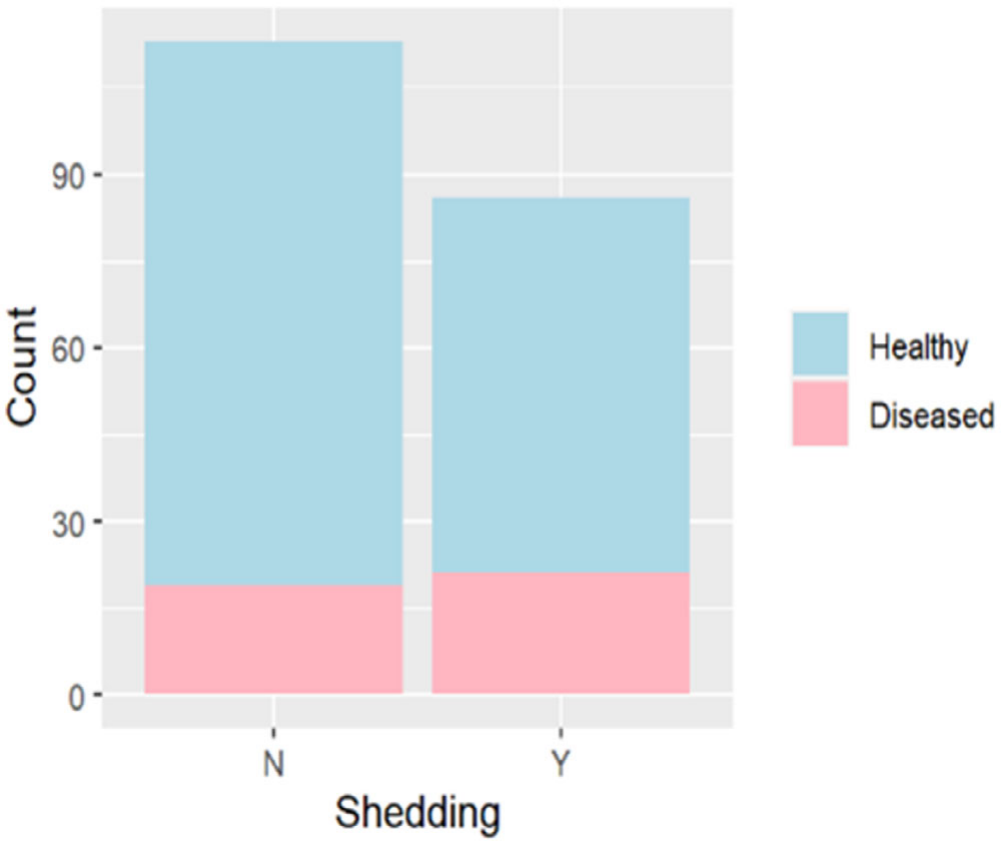
Frequency of shedders (Y) and non‐shedders (N) showing upper respiratory tract disease.

**FIGURE 5:**
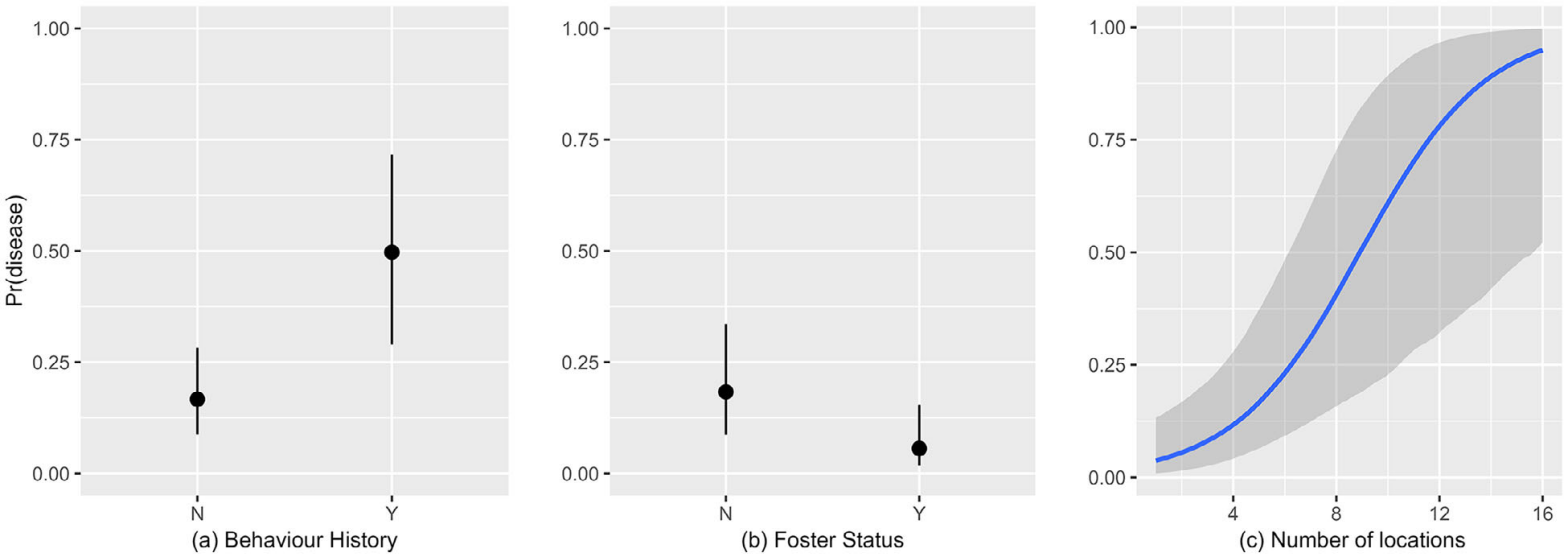
Modelled conditional effects of (a) behavioural intervention, (b) being fostered on clinical upper respiratory tract disease and (c) number of locations (calculated at average values for all covariates). Pr(disease) = probability of disease.

While shedding animals were 40% more likely to show clinical disease compared to non‐shedders (Figure 4), model estimates showed large uncertainties. Only an animal's behavioural status (OR 5.0, 95% CI 2.4‒11), foster status (OR 0.27, 95% CI 0.09‒0.78) and the number of locations (OR 1.51, 95% CI 1.19‒1.93) showed a majority of the posterior mass away from 1, suggesting a strong possibility of their effect on onset of disease (Figure 5).

**FIGURE 6:**
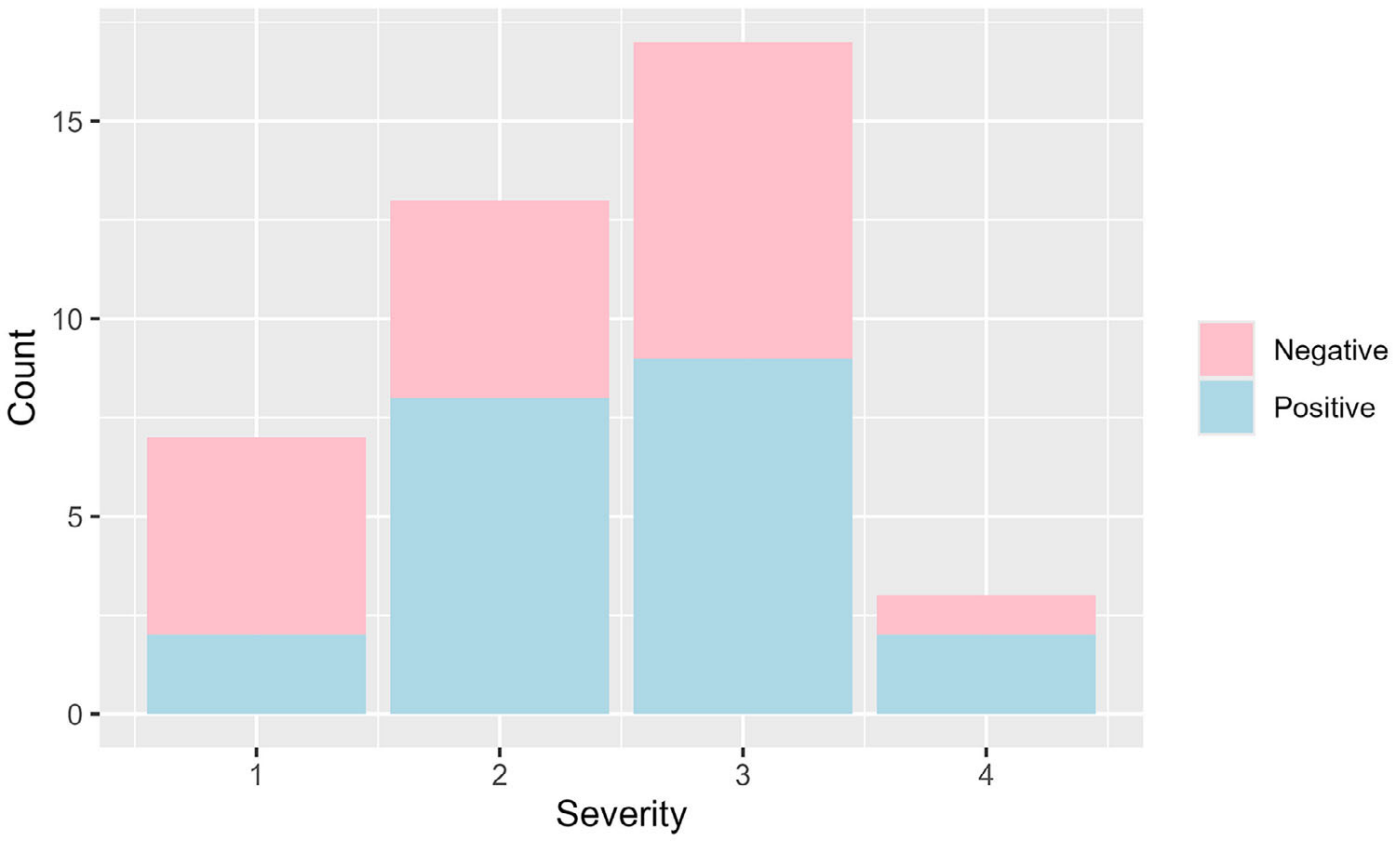
Frequency of each severity grade showing positive and negative PCR result for upper respiratory tract pathogens.

**FIGURE 7:**
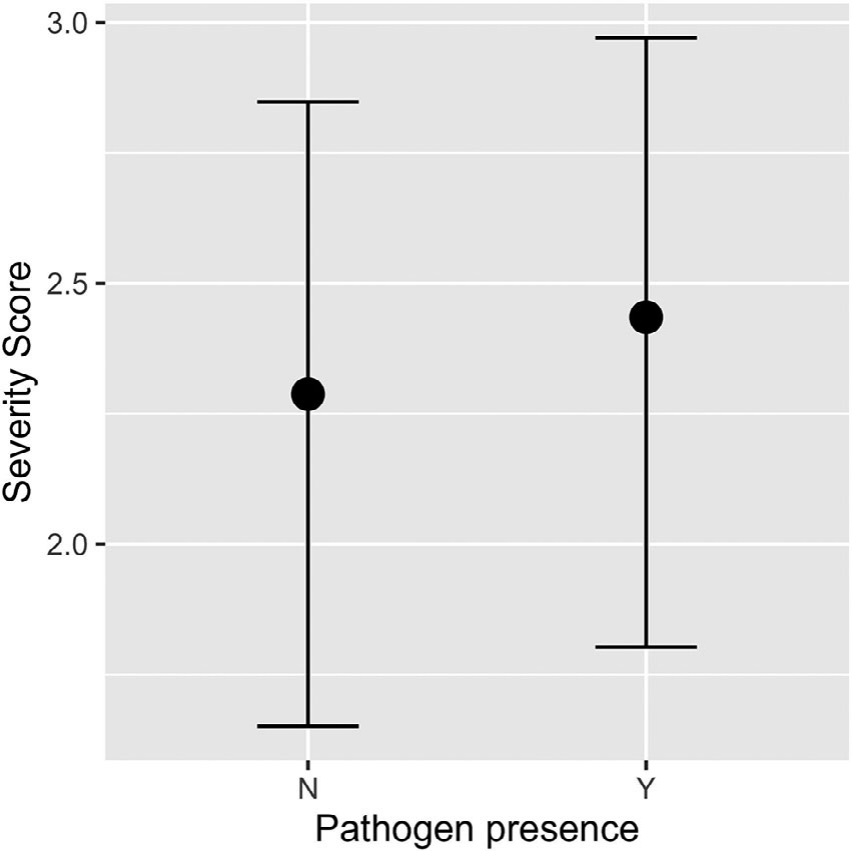
Modelled conditional effects of pathogen presence on disease severity scores (calculated at average values for all covariates).

While larger numbers of shedding animals had higher severity scores (Figure 6), the modelled association of severity with pathogen presence (Figure 7) was mostly positive but again with large uncertainties (OR 1.51, 95% CI 0.46‒4.94). The number of locations (OR 1.82, 95% CI 1.31‒2.65) and presence of multiple pathogens (OR 2.52, 95% CI 0.61‒10.11) were positively associated with severity.

### Risk factors associated with euthanasia as a final outcome

**FIGURE 8:**
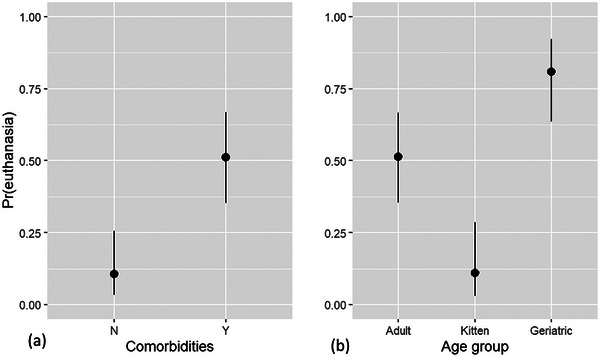
Modelled conditional effects of (a) comorbidities and (b) age bracket on probability of euthanasia (calculated at average values for all covariates). Pr(euthanasia) = probability of euthanasia.

While shedding pathogens did not affect shelter outcome, there was association of the presence of comorbidities (OR 8.8, 95% CI 3.5‒25) and age on the probability of euthanasia. Compared to adults, kittens were highly unlikely (OR 0.12, 95% CI 0.03‒0.36) and geriatric animals were more likely (OR 4.1, 95% CI 1.8‒10) to be euthanased (Figure 8).

Since current age classification guidelines do not reflect the software's automated boundaries,^34^ a sensitivity analysis was done, which found no meaningful change in results with differing classification boundaries. All estimated regression coefficients and their standard errors are provided in Supporting Information S6.

## DISCUSSION

While 86 incoming animals (43%) were shedding pathogens at the time of entry into the shelter, less than one‐quarter of these went on to develop clinical disease. Almost the same number of non‐shedders also developed disease, indicating no likely association between pathogen presence and subsequent disease. There was mild increase in the severity of disease in shedders. Past studies have found contrasting results to our study. A strong likelihood of association was found in owned cats between presence of FHV and FCV and signs such as sneezing, nasal discharge, coughing, pyrexia and anorexia.^35^ Similar associations have been reported in a diverse cohort of cats with FHV,^14^, ^36^ *B. bronchiseptica*,^37^ FCV^38^, ^39^, ^40^ and *C. felis*.^39^, ^41^, ^42^

Our findings suggest that routine PCR testing is unlikely to serve as an accurate tool to diagnose or predict clinical infection in shelter settings. Instead, diagnosis should rely on veterinary assessment and response to treatment. Additionally, isolated cases of cats becoming clinically infected and shedding viruses introduced through live vaccinations have been reported.^43^, ^44^, ^45^ Therefore, it is possible that a positive PCR result may reflect recent vaccination rather than a true infection. Moreover, since FHV can be shed in clinically healthy animals, high test prevalence may not necessarily reflect true prevalence of clinical disease.^46^, ^47^

Similar to our study, a previous shelter‐based study reported no association between pathogens and the entry and development of subsequent disease,^5^ concluding that while introduction of pathogens may not be entirely avoidable, the incidence of disease can be well controlled by lowering levels of stress through targeted husbandry practices. One example of targeted management that our shelter practiced was placing selected animals into foster care.

The link between stress and URT infection is well established, especially in the context of viral recrudescence,^4^, ^5^, ^48^, ^49^ reinforcing the importance of balancing mental or emotional health for reducing pathogen transmission within shelters. Our DAG showed several environmental stressors subsequently affecting immunity. While some of these are not easily modifiable (such as pre‐existing illness), others can be influenced by shelter operations. For example, cats that were not placed into foster care were almost four times as likely to get clinical disease. While a previous study by the authors revealed that this relationship to be reversed,^29^ reasons for this discrepancy could be the limited scope to incorporate temporality of fostering with disease incidence. In the present study, most animals first developed clinical disease and then found foster placement.

While shelter staff make a concerted effort to place animals into foster, less than a quarter of cats in our study were fostered. Hence a possible strategy to reduce disease could be to re‐direct priorities and resources into expanding the foster programme, subsequently having a domino effect on other metrics such as reduction in LOS, lowering the number of housing locations per animal and increasing adoption rates.

Behavioural intervention included a combination of medical anxiolytics, gentle, positive interactions and housing in an area with lower foot traffic. A PAF value of roughly +0.10 over a 50‐day period shows that if the need for behavioural rehabilitation was removed, we could expect a 10% decrease in cases. The PAF estimate shows a slight decrease over duration of shelter stay, indicating that early behavioural intervention would be more impactful.

While under 20% of animals needed behavioural support, it is likely that those that did, were under sustained stress from the time of entry (and possibly pre‐entry) until they were identified and started on intervention. Although cats needing behavioural support showed a strong association with disease, we believe that this programme played an important role in limiting even greater disease burden. One study found that daily positive interactions with animals that were in a state of low stress not only helped maintain that state, but animals that did not get these interactions showed increased pathogen shedding as well as behaviour indicating regression to a more anxious state.^50^ This demonstrates the importance of environmental and managemental interventions (such as positive human interactions) for cats housed in close confinement in shelters.

Geriatric cats and kittens showed greater disease severity compared to adults, although kittens showed a delayed time to onset of disease. While it is possible that geriatric cats may have had longer to build immunity through previous infection or vaccinations, some possible reasons for our results could include host‐related factors (such as concurrent infection, poor living conditions and chronic stress) rendering vaccinations less effective.^6^ Additionally, if these cats were already carriers at the time of vaccination, they may not undergo adequate antigenic stimulation and subsequent mounting of an immune response.^51^

Maternal immunity towards feline URT infection is thought to wane by 4‒14 weeks of age^52^; the level of immunity is dependent on the immune status of the mother. The majority of kittens were brought into the shelter with no known history; therefore, we believe that it is unlikely that these kittens were provided with effective protection through maternal antibodies. It is possible that physical segregation of younger animals from the main housing areas and faster foster placement played a role in protecting some of these cats from infection. Of the 40 diseased cats, since only six were geriatric and four were kittens, small sample sizes may not have yielded representative associations.

While four cats died of natural causes, more than one‐third of animals were euthanased—10 were euthanased due to complications with feline URT disease. Our analysis did not show substantial effect of either the presence of pathogens or disease on an animal's chance of being euthanased. Only an animal's age and the presence of comorbidities (such as severe injuries, positive FIV test result and chronic illness) showed a strong association with likelihood of euthanasia. These findings likely reflect the shelter's policies underpinned by cost‒benefit and quality of life analyses (especially for cats needing long convalescence or lifelong treatment) and the unsuitability of such cats to be adopted into new homes.

Animal shelters are an incredibly complex environment, with a dynamic combination of factors impacting final outcomes. Environmental factors such as housing density, housing size, quality of enrichment and hygiene have been found to influence prevalence of disease.^35^, ^37^ Both LOS and movement between locations are emerging as an important metric in assessing the success of a shelter's operations.^5^, ^53^ Most animals in our study spent less than a month in the shelter (although the longest LOS was over 6 months) and were housed in an average of four locations.

While LOS did not show strong association, the number of locations was positively associated with both presence and severity of disease. Studies have shown that increased LOS has a temporal (non‐causal) relationship with increased disease prevalence.^1^, ^10^, ^29^ The longer a cat stays in a shelter, the greater its chances of getting infected with common diseases endemic in shelters.^11^ While the effect of moving animals has not been specifically assessed before, some association between stress from rehousing and reactivation of FHV has been seen.^54^, ^55^ Besides causing increased stress, moving animals are also believed to play a role in elevated risk of disease transmission: cats that spent a longer time in transport had an increased frequency of URT disease,^10^ which is consistent with other studies linking stress, viral recrudescence and disease transmission due to relocation. One study found that greater than 8 ft^2^ of floor space per cat was associated with lower URT infection compared to smaller cages.^5^ It is possible that cage size may have played a role in URT incidence because several cages at our shelter did not meet this criterion; however, this was not tested in our study.

The relationship of disease with LOS, foster care and number of housing locations was captured in our DAG. However, temporal feedback loops preclude obtaining an unbiased estimate of their causal effect. Once a cat has developed clinical signs, the time and hospitalisation needs increased overall LOS as well as number of locations (or the cat was euthanased, reducing LOS). There is also a temporal relationship between illness and being prioritised for foster: cats with recurrent or non‐responsive signs are prioritised, to increase their chance of recovery. These relationships are likely continually bidirectional (where LOS, foster status and number of locations affect risk of clinical disease, which in turn affects LOS, chance of foster placement and number of locations), rendering associations non‐causal, especially where the feedback occurs more quickly than the time scale of the collected data.^56^

It is likely that small sample sizes precluded unbiased causal estimates for certain parameters—only two cats were seized due to cruelty complaints, and only four kittens and six geriatric cats developed clinical disease. Previous work by the authors demonstrated relationships between disease classification and neuter status, age and source of animal, some of which were not demonstrated in our study.^29^

While PCR results for five URT pathogens were differentiated, we used a binary PCR result (positive or negative) in our study. Since most shelters do not have the resources to routinely conduct PCR tests, identifying specific pathogen associations may be of limited use to shelter operations and was outside the scope of this study. However, this method may be of use for further studies with a focus on understanding the pathogenesis of specific pathogens.

While previous research has shown that size of enclosures has considerable impact on infection risk, this was outside the scope of our study. While it was common to see incoming animals shedding pathogens on entry, shedding was not associated with subsequent development of clinical disease, rendering positive PCR results not necessarily predictive of true clinical infection. Animal‐based characteristics and shelter operations are more influential risk factors. Expanding the foster programme, reducing movement between locations and prompt commencement of the behaviour rehabilitation programme have the potential to substantially reduce prevalence of clinical feline URT disease within this shelter.

## AUTHOR CONTRIBUTIONS

*Supervision, study design and draft editing*: Nicholas Clark and Mandy Paterson. *Study design, primary draft writing, statistical analysis, field research, data collection and handling*: Uttara Kennedy. *Statistical analysis supervision and draft editing*: Nicholas Clark and Mark Stevenson. *Laboratory testing and reporting and draft editing*: Susan Jaensch and Doug Hayward.

## CONFLICTS OF INTEREST

The authors declare they have no conflicts of interest.

## ETHICS STATEMENT

The authors confirm that the ethical policies of the journal, as noted on the journal's author guidelines page, have been adhered to. The protocol and procedures for this research were approved by the Animal Ethics Committee at University of Queensland (AE000528).

## Supporting information



Supporting Information

## Data Availability

The data that support the findings of this study are available on request from the corresponding author. The data are not publicly available due to privacy or ethical restrictions.
