# Supplementary material for: Prevalence of feline upper respiratory tract pathogens and risk factors for clinical disease and final outcomes in an RSPCA shelter in Queensland, Australia
Source: Vet Rec Open. 2024 Dec 6;11(2):e270001. doi: 10.1002/vro2.70001 (PMC11622150; doi:10.1002/vro2.70001)
Supplement: Supplementary file 1 — Supporting Information [file VRO2-11-e270001-s001.pdf]

Supporting Information

S1.1 - A typical shelter enclosure for (a) individual and (b) group housing

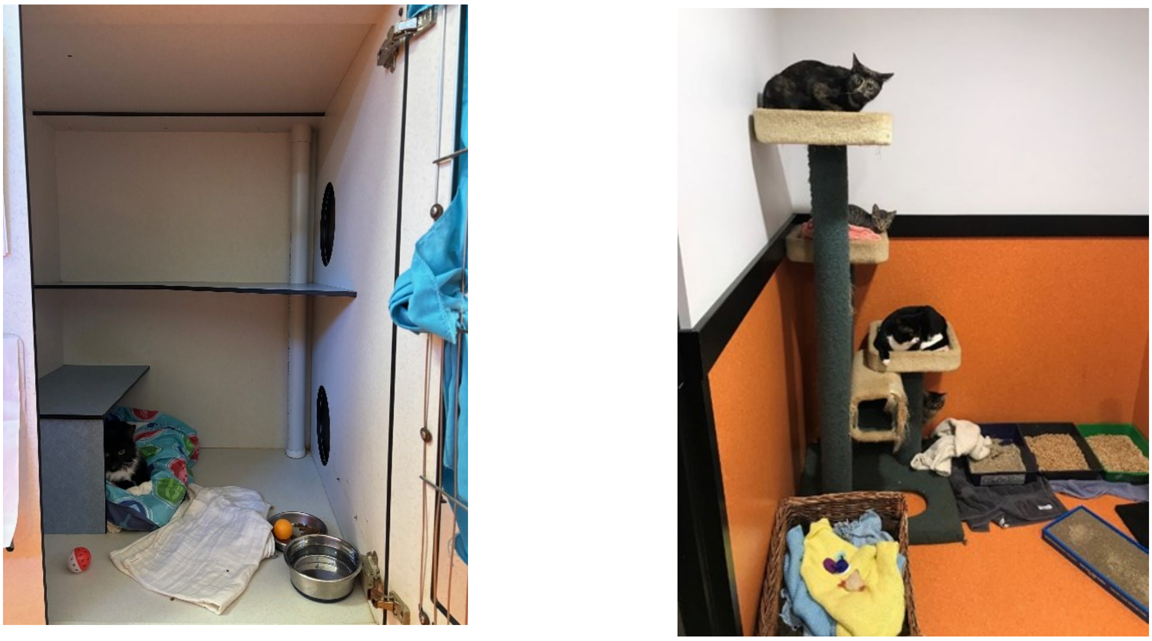

Each enclosure was furnished with soft bedding of towels, metal or plastic feeding and watering bowls and a litter tray. Age-appropriate commercial food was provided twice a day, or as per the individual animal's requirement. The shelter followed strict biosecurity measures, including (1) spot cleaning cages daily (removing debris and spraying cages with Pet Forte Pro® @1:60 with a foam applicator left for 10 minutes), (2) washing toys, bedding, litter trays and bowls; (3) disinfection of cages between cats with the same regime repeated twice. Animal care staff wore gloves and protective gowns during cleaning of cages and washed their hands with a foaming alcohol handrub following contact with an animal or its excreta.

S1.2 - Selection Criteria

1. All cats estimated to be over six weeks of age were eligible for inclusion.
2. If a large group of animals (>5) were brought from a single place of residence, then only a few of these animals were chosen to prevent over-representation.
3. Cats transferred from another RSPCA shelter were excluded.
4. Cats returned to the shelter less than two weeks from being adopted from an RSPCA affiliated shelter or pet store were excluded.

S2.1 - Standard Operating Protocol for feline conscious restraint for oropharyngeal and conjunctival swabbing

I. OBJECTIVE

To describe the safe and humane way to gently restrain cats for conscious oropharyngeal and conjunctival swabbing.

II. COMMENTS / RECOMMENDATIONS

- Carefully monitor the cat's demeanour to avoid cat scratches, bites or needle stick injuries.
- This procedure should not take longer than five minutes per animal in total.

The diagnostic quality of the samples collected may be impacted if contaminated with fluorescein stain, eye ointments, or other ocular/ nasopharyngeal medications recently applied to the animal. If collecting epithelial cells, diagnostic quality of samples may also be impacted if there are large amounts of ocular or nasopharyngeal discharge at the sample site. Store swabs as per specific laboratory instructions until they can be sent to the laboratory.

III. EQUIPMENT

1. Dry, sterile cotton swabs (preferably those with a flexible plastic shaft).
2. Individual, sterile sample transport containers (containing an appropriate transport media for the swab).
3. Clean, dry towel for restraint.
4. Clean, dry surface (table or floor).
5. Feliway spray (if available)

IV. PROCEDURE

1. Prepare area to ensure that there is a rubber mat or towel in between the cat and table or floor of the enclosure. Preparation should include spraying the towel with Feliway, if available.
2. Ensure all your equipment is ready to go, and that the sampling area is secure.
3. The handler should safely remove the cat from the cage or condo. Ensure you are using correct handling techniques.
4. The handler should gently place the cat on the table or floor.
5. To start have the cat strategically placed in the centre of the towel and ensure that there is roughly 10 cm of towel in front of the cat.
6. The cat can be standing, sitting or lying down.
7. The handler should keep one hand on the cat while picking up the towel at the front of the cat. Bring the towel back enclosing the front limbs and wrap around the cat's neck. Allow for one to two finger tightness between the cat's neck and towel.
8. While holding the towel around neck, the handler uses the other hand to wrap towel firmly around the cat's body, ensuring all limbs have been included. Tuck the edges in under the cat. Then wrap the other side and again tuck edges in under the cat.
9. Check the tightness of the towel wrap and fix any issues up.
10. Restrain the cats head by the handler placing a thumb at the base of the cat's ear or skull on one side and fingers at the base of the cat's ear or skull on the other. Ensure fingers are not wrapped around the cat's throat or in the way of the person swabbing.
11. The handler should restrain the cat's forelimbs below the elbow (between radius and ulna).
12. The cat is restrained between handlers' body and forearm.

13. The person collecting the swabs then obtains the conjunctival and pharyngeal swabs.
    (a) Pharyngeal samples - gently tilt the face upwards and open the mouth with one hand, then with the other hand insert and then gently roll a sterile dry swab across the desired laryngopharyngeal area.
    (b) Conjunctival samples - reflect the lower eyelid with one hand, then use the other hand to gently roll a sterile dry swab across the ventral conjunctival fornix of one, or both eyes.

    Generally collecting samples from multiple sites increases test sensitivity, however, if only a single site can be collected, dependent on the sampling purpose, a pharyngeal swab is usually preferred.
14. The swabs should be placed into individual sterile sample transport containers. Swab stems can be shortened using sterile scissors (if required to fit within the containers).
15. Label the containers and store them as per specific laboratory instructions prior to sending to the laboratory for testing.

    Generally, for short term storage, samples require standard refrigeration (0-4°C).

S2.2 - Molecular analysis methods

Swabs were thawed then extracted on a Roche MagNA Pure 96 (Roche, Basel, Switzerland) using the Universal Pathogen Protocol including a DNA internal control. In brief, proprietary magnetic glass particles and MagNA Pure 96 Kits were used. DNA and RNA bind to the glass surface of the magnetic glass particles in the presence of isopropanol and high concentrations of chaotropic salts, which remove water from hydrated molecules in solution. Polysaccharides and proteins do not bind to the beads and are removed by sequential washing steps. Once bound to the surface of the magnetic glass particles, the nucleic acids are separated from the solution with magnets that are placed in between the wells of the processing cartridge. The liquid phase is then aspirated and removed. Pure nucleic acids were eluted from the beads by applying low-salt conditions and heat.

Extracted DNA samples then underwent two-step PCR utilising an automated AusDiagnostics High-Plex analyser and using a commercial kit targeting canine and feline respiratory pathogens (AusDiagnostic SA Respiratory). High-Plex analysis performed multiplexed tandem Polymerase Chain Reaction (MTPCR) employing two sequential PCR steps. Step 1 was a short-multiplexed pre-amplification reaction using primers homologous to all targets in the kit utilised. Step 2 contains separate primer pairs for every target offered by the panel, designed to be nested inside Step 1 primers, increasing the sensitivity and specificity of the assay, whilst diluting out potential inhibitory and cross-reacting substances. The Step 2 reaction resulted in fluorescence emission once reagents were intercalated in double-stranded DNA. The fluorescence increase during amplification was monitored throughout the reaction time and analysed by MT Analysis Software.

S3 Variables as recorded on the RSPCA Queensland (Qld) database

1. Age - If the animal's historical information includes a recorded age (e.g. on microchip details or previous veterinary records) this was recorded as an animal's age at entry. Where no previous information was available, an animal's age was estimated by RSPCA Qld veterinary staff, by means of thorough physical examination. In cases where no age was recorded by the end of the animal's time at the shelter, age was estimated from clinical history by the primary author (UK). For all models, age was converted into a factored variable: animals aged < four months (Kitten), animals aged between four months and eight years (Adult) and animals > eight years (Geriatric)

2. Gender - An animal's gender was recorded on entry by RSPCA Qld staff as male or female, based on physical examination. If this information was not recorded by the time the animal had left the shelter/died/ben euthanised, this field was recorded as NA.

3. Animal Source - This variable defines the circumstance under which an animal entered the RSPCA. For our study, the four most common sources were included: *Stray*- any animal thought to be stray or unowned at time of entry, *Surrender*- any animal surrendered to the shelter by a proven owner, *Ambulance*- any animal brought in via the RSPCA Qld ambulance service (sometimes but not always injured or ill) and *Other*- all other circumstances including animals seized by RSPCA inspectors due to welfare concerns, adopted animals being returned, transfers from other shelters, off-spring born in care, animals dead on arrival, bequeathed animals and animals temporarily boarding due to enrolment in the domestic violence program.

4. Neuter/Desex status on entry - Each animal was checked upon entry for evidence of previous neutering. In the case of males, this was ascertained through physical examination by RSPCA Qld staff for the absence of testicles. In the case of females, this was ascertained through the presence of an ear tattoo and/or desex scar.

5. Pathogen presence - As determined by each animal's PCR results.

6. Coinfection - The presence of multiple pathogens on PCR results.

7. Foster Status - Whether an animal went out into foster care for any length of time. This information was extracted from the database after the animal had left the shelter/died/been euthanised.

8. Length of Stay (LOS) - The number of days that each animal spent within the shelter.

9. Comorbidities - Whether an animal entered the shelter with existing conditions or developed conditions that needed treatment. These include systemic illness, traumatic injuries, toxicities and co-infections (e.g. FIV). Mild conditions like low grade dental disease or presence of fleas were excluded.

10. Behavioural Status Animals that were unable to be closely handled or showing overt signs of stress or anxiety, were enrolled in the shelter's behaviour rehabilitation program. These animals were closely managed (medically and behaviourally) by the rehabilitation team and often segregated from the general population until they were able to leave the program. These animals were assigned a behavioural sub-status, enabling us to extract information about entry into the rehabilitation program. This information was extracted from the database after the animal had left the shelter/died/been euthanised.

11. Number of locations - Animals were often moved within or between shelters for various reasons (e.g. pre-operative and post-operative areas during surgical procedures, quarantine or hospital locations in case of illness, between pens due to cleaning protocols, space restrictions or outbreaks). The number of locations each animal spent time in was extracted from the database after the animal had left the shelter/died/been euthanised.

12. Final outcome The final status of an animal at the time of first leaving shelter care/dying/being euthanised after its entry date was extracted from the database.

13. Presence/Absence of feline URT clinical disease - Determined by manual annotation of veterinary records by UK.

14. Severity Score - Determined by manual annotation of veterinary records by UK, using a scoring chart (Grade 1-4). The first day that each score was reached was recorded.

S4 - Severity Score Chart

Grade 4 Euthanised due to non-responsiveness to treatment for cat flu.

Grade 3 [If there was a diagnosis by the veterinarian of flu (see word list A) OR If any signs (see word list B) were observed as thought to be related to cat flu] AND [An antibiotic was commenced as treatment for flu OR Systemic anti-viral medication was commenced]

Grade 2 [If there was a diagnosis by the veterinarian of flu (see word list A) OR If any signs (see word list B) were observed as thought to be related to cat flu] AND [Appetite stimulant treatment OR steaming OR short-term NSAID treatment was commenced as treatment for flu OR topical treatment was given for cat flu] AND [No antibiotics or antivirals were prescribed for cat flu].

Grade 1 [If any signs (see word list B) were observed as thought to be related to cat flu] AND [No treatment was given for cat flu.]

Word list A - 'cat flu' or 'URI' or 'feline upper respiratory tract infection' or 'CF', 'herpes virus', 'herpes', 'calici virus', 'calicivirus', 'calici', 'chlamydia', 'mycoplasma' or 'bordetella'

Word list B - 'chemosis', 'ocular discharge', 'blepharospasm', 'squinting' and 'conjunctivitis', 'watery eyes', 'ocular discharge', 'decreased appetite', 'decreased food intake', 'anorexia', 'congestion', 'snuffling', 'nasal discharge', 'sneezing', 'coughing', 'open mouth breathing', 'OMB' or 'hard swallow'

S5 - Prior predictive checks

Prior parameter distributions were used to simulate hypothetical data. The resulting priors were weakly informative, encouraging regularised posterior estimates. β coefficient and standard deviation priors, representing effect size and variation, were chosen based on a normal distribution with mean of 0 and standard deviation of 1, to reflect our belief in effect size and variation no greater than exp(1) = 2.7 in either direction. Intercept priors were chosen to reflect our prior belief of expected disease prevalence and euthanasia rates between 19% and 24%, represented by a normal distribution as seen in the figure below, with a mean of -1.4 and standard deviation 0.7 on a logit scale.

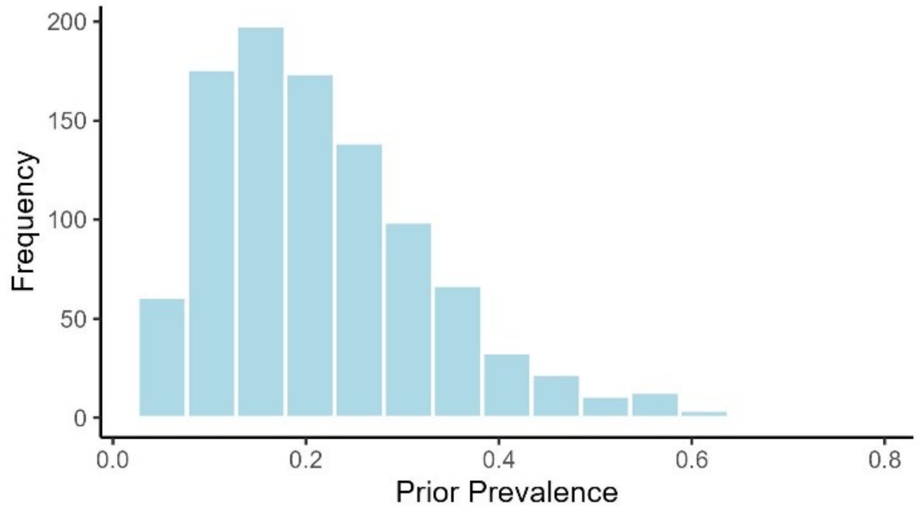

Figure S1 Distribution of prior infection prevalence and euthanasia rate

S6 - Regression models for presence of clinical upper respiratory tract (URT) signs, severity and final outcome

- Clinical URT signs/final outcome by Pathogen presence, Neuter status, Number of locations, Length of stay (LOS) and Foster status (Adjusted).

$$logit(\pi) = \alpha + \alpha_{source} + \beta_{pathogen} * \boldsymbol{pathogen} + \beta_{desex} * \boldsymbol{desex} + \beta_{comorbidities} * \boldsymbol{comorbidities} + \beta_{behaviour} * \boldsymbol{behaviour} + \beta_{los} * \boldsymbol{los} + \beta_{locations} * \boldsymbol{locations} + \beta_{fostered} * \boldsymbol{fostered} + \beta_{coinfected} * \boldsymbol{coinfected} + \beta_{age} * \boldsymbol{age\_bracket}$$

- Clinical URT signs/final outcome by Multiple infections (Adjusted):

$$logit(\pi) = \alpha + \alpha_{source} + \beta_{desex} * \boldsymbol{desex} + \beta_{comorbidities} * \boldsymbol{comorbidities} + \beta_{behaviour} * \boldsymbol{behaviour} + \beta_{los} * \boldsymbol{los} + \beta_{locations} * \boldsymbol{locations} + \beta_{fostered} * \boldsymbol{fostered} + \beta_{coinfected} * \boldsymbol{coinfected} + \beta_{age} * \boldsymbol{age\_bracket}$$

- Clinical URT signs/final outcome by Source (Adjusted):

$$logit(\pi) = \alpha + \alpha_{source} + \beta_{pathogen} * \boldsymbol{pathogen} + \beta_{comorbidities} * \boldsymbol{comorbidities} + \beta_{behaviour} * \boldsymbol{behaviour} + \beta_{los} * \boldsymbol{los} + \beta_{locations} * \boldsymbol{locations} + \beta_{fostered} * \boldsymbol{fostered} + \beta_{coinfected} * \boldsymbol{coinfected} + \beta_{age} * \boldsymbol{age\_bracket}$$

- Clinical URT signs/final outcome by Age bracket (Adjusted):

$$logit(\pi) = \alpha + \alpha_{source} + \beta_{pathogen} * \boldsymbol{pathogen} + \beta_{comorbidities} * \boldsymbol{comorbidities} + \beta_{behaviour} * \boldsymbol{behaviour} + \beta_{coinfected} * \boldsymbol{coinfected} + \beta_{age} * \boldsymbol{age\_bracket}$$

- Clinical URT signs/final outcome by Comorbidities and Behavioural intervention (Adjusted):

$$logit(\pi) = \alpha + \alpha_{source} + \beta_{pathogen} * \boldsymbol{pathogen} + \beta_{desex} * \boldsymbol{desex} + \beta_{comorbidities} * \boldsymbol{comorbidities} + \beta_{behaviour} * \boldsymbol{behaviour} + \beta_{coinfected} * \boldsymbol{coinfected} + \beta_{age} * \boldsymbol{age\_bracket}$$

Table S6.1 Coefficient estimates for variables associated with presence of clinical URT signs

Variable	Odds Ratio (Adjusted)	Credible Interval
Pathogen presence (Y)	1.35	0.58, 3.10
Neuter status (Y)	0.86	0.33, 2.10
**Number of locations**	**1.51**	**1.19, 1.93**
Comorbidities (Y)	1.22	0.56, 2.80
**Behavioural intervention (Y)**	**4.99**	**2.39, 10.98**
**Foster status (Y)**	**0.27**	**0.09, 0.78**
Multiple infections (Y)	1.28	0.46, 3.36
Age bracket (Kitten)	0.73	0.26, 1.91
Age bracket (Geriatric)	0.58	0.23, 1.37

(**bold** indicates significance)

Table S6.2 Coefficient estimates for variables associated with severity of clinical URT signs

Variable	Odds Ratio (Adjusted)	Credible Interval
Pathogen presence (Y)	1.51	0.46, 4.94
Neuter status (Y)	0.99	0.28, 3.51
**Number of locations**	**1.82**	**1.31, 2.65**
Comorbidities (Y)	0.70	0.22, 2.36
Behavioural intervention (Y)	0.65	0.21, 2.02
Foster status (Y)	0.61	0.13, 2.59
Multiple infections (Y)	2.52	0.61, 10.11
Age bracket (Kitten)	1.78	0.44, 7.42
Age bracket (Geriatric)	2.58	0.64, 9.40

(**bold** indicates significance)

Table S6.3 Coefficient estimates for variables associated with shelter outcome (euthanised versus adopted)

Variable	Odds Ratio (Adjusted)	Credible Interval
Pathogen presence (Y)	1.16	0.43, 3.07
Neuter status (Y)	0.92	0.31, 2.59
**Comorbidities (Y)**	**8.77**	**3.55, 25.14**
Behavioural intervention (Y)	1.05	0.35, 3.28
Foster status (Y)	0.65	0.16, 2.49
Multiple infections (Y)	2.46	0.69, 8.92
**Age bracket (Kitten)**	**0.12**	**0.03, 0.36**
**Age bracket (Geriatric)**	**4.11**	**1.80, 10.19**

(**bold** indicates significance

Where LOS = Length of Stay; $\alpha_{source} \sim Normal(\mu_{source}, \sigma_{source})$
